# Compartmented neuronal cultures reveal two distinct mechanisms for alpha herpesvirus escape from genome silencing

**DOI:** 10.1371/journal.ppat.1006608

**Published:** 2017-10-26

**Authors:** Orkide O. Koyuncu, Margaret A. MacGibeny, Ian B. Hogue, Lynn W. Enquist

**Affiliations:** 1 Department of Molecular Biology, and Princeton Neuroscience Institute, Princeton University, Princeton, NJ, United States of America; 2 School of Life Sciences, and Biodesign Institute Center for Immunotherapy, Vaccines and Virotherapy, Arizona State University, Tempe, AZ, United States of America; Duke University Medical Center, UNITED STATES

## Abstract

Alpha herpesvirus genomes encode the capacity to establish quiescent infections (i.e. latency) in the peripheral nervous system for the life of their hosts. Multiple times during latency, viral genomes can reactivate to start a productive infection, enabling spread of progeny virions to other hosts. Replication of alpha herpesviruses is well studied in cultured cells and many aspects of productive replication have been identified. However, many questions remain concerning how a productive or a quiescent infection is established. While infections *in vivo* often result in latency, infections of dissociated neuronal cultures *in vitro* result in a productive infection unless lytic viral replication is suppressed by DNA polymerase inhibitors or interferon. Using primary peripheral nervous system neurons cultured in modified Campenot tri-chambers, we previously reported that reactivateable, quiescent infections by pseudorabies virus (PRV) can be established in the absence of any inhibitor. Such infections were established in cell bodies only when physically isolated axons were infected at a very low multiplicity of infection (MOI). In this report, we developed a complementation assay in compartmented neuronal cultures to investigate host and viral factors in cell bodies that prevent establishment of quiescent infection and promote productive replication of axonally delivered genomes (i.e. escape from silencing). Stimulating protein kinase A (PKA) signaling pathways in isolated cell bodies, or superinfecting cell bodies with either UV-inactivated PRV or viral light particles (LP) promoted escape from genome silencing and prevented establishment of quiescent infection but with different molecular mechanisms. Activation of PKA in cell bodies triggers a slow escape from silencing in a cJun N-terminal kinase (JNK) dependent manner. However, escape from silencing is induced rapidly by infection with UVPRV or LP in a PKA- and JNK-independent manner. We suggest that viral tegument proteins delivered to cell bodies engage multiple signaling pathways that block silencing of viral genomes delivered by low MOI axonal infection.

## Author summary

Alpha herpesvirus infections stay life-long in infected human and animal hosts`nervous systems in a silent state ready to reactivate upon various stress signals. Remarkably, infection of epithelial cells with these viruses results in productive infection whereas infection of peripheral nervous system neurons results in non-productive silent infection (i.e. latency) in the natural hosts. More interestingly, infection of dissociated peripheral neurons in culture also results in productive infection unless DNA replication inhibitors are used. To study the molecular mechanisms of escape from latency, we used primary neurons cultured in compartmented tri-chambers. By this way, we recapitulated the natural route of infection by infecting axons with low dose of virus which resulted in a silent infection in a small number of neuronal cell bodies without the use of any inhibitors. Using these cultures, we developed a new complementation assay to investigate the molecular signals leading to escape from latency and establishment of productive infection. We found two different mechanisms to escape from latency: Cellular stress-mediated slow route and viral tegument mediated-fast route. Furthermore, we showed that the stress-mediated pathway requires protein kinase A and c-Jun N-terminal kinase activity while the viral tegument-mediated fast escape does not require these host cell kinase activities. We also concluded that a general response to DNA virus infection or presence of excess herpesviral genomes in the nucleus to saturate silencing complexes is not enough to escape from latency. Induction of a productive infection requires presence of tegument proteins or activation of PKA and JNK pathway.

## Introduction

In an infected host, herpesviruses initiate a productive infection cycle in a variety of cell types, but in some cells, they can establish a quiescent or latent infection [1]. These latent infections may be reactivated resulting in a productive infection. The current effective antiviral drugs suppress productive replication, but there are no treatment modalities to block the establishment of quiescent infections. Alpha herpesviruses, including herpes simplex virus (HSV; human herpesvirus 1 and 2), varicella zoster virus (VZV; human herpesvirus 3), and pseudorabies virus (PRV; suid herpesvirus 1), establish life-long latency in the peripheral nervous system (PNS) of their natural hosts. PNS neurons are terminally differentiated, and the association of their cell bodies with peripheral organs is mediated by axons that extend long distances. As a result, axons reside in a different milieu than their cell bodies. This architecture indeed affects the mode of alpha herpesvirus infections, but is difficult to recapitulate *in vitro*. Much of the research on herpesvirus latency and reactivation involves animal models, but *in vitro* models have been developed to provide a more reductionist approach for analysis of the molecular biology of alpha herpesvirus latency [2–4]. These studies have revealed several important findings, including how continuous neuronal signaling is required to maintain latency, and how histone modifications trigger general transcriptional activation of silenced viral promoters. Most of these *in vitro* models use dissociated neuron cultures where axons and cell bodies are not physically or fluidically separated. In addition, to establish a quiescent infection, isolated PNS neurons must be pretreated with interferon or replication inhibitors (e.g. acyclovir) to block the initial productive infection.

In this report, we used the modified Campenot tri-chambers to physically and fluidically separate axons from their cell bodies. By culturing primary PNS neurons in these chambers, we were able to establish reactivateable quiescent infections by PRV in the absence of any replication inhibitor or cytokine treatment [5]. Quiescent infections were established only when isolated axons were infected with infectious virions at a very low MOI (MOI of 0.01 pfu/cell). Here we use this simplified experimental system to establish a quiescent infection (i.e. latency) and to investigate the factors regulating escape from silencing. Our assay is a complementation assay in which cell bodies are exposed to different treatments at the same time that axons are infected with a very low MOI infection of a PRV recombinant that expresses mRFP-VP26 (PRV 180) to enable visualization of productive replication in cell bodies.

First, we found treatments that elevated cyclic adenosine monophosphate (cAMP) levels (e.g. forskolin) in the cell bodies were sufficient to promote productive replication of PRV 180. Such escape from silencing was mediated by protein kinase A (PKA) activation and required cJun N-terminal kinase (JNK). We also found that applying UV inactivated PRV virions (UVPRV) to cell bodies very efficiently promoted escape from silencing. Infection of cell bodies with UV inactivated virus mutants that could not engage cell surface receptors (gD null) or could not enter cells by membrane fusion (gB null) did not promote escape from silencing. Importantly, activating PKA in cell bodies took longer to promote escape from silencing than did exposing cell bodies to UVPRV.

We then focused on understanding how inactivated PRV particles could induce such rapid escape from silencing. An important discovery came from complementation studies with light particle (LP) preparations generated after infection by a UL25 null PRV mutant that produced LP but no infectious virions. Herpesvirus infected cells typically produce light particles together with infectious virions [6–8]. LP contains no capsids or viral genomes, but carries outer tegument proteins and most of the viral envelope proteins. In our complementation assay, cell body infection with LP promoted efficient PRV escape from silencing. Moreover, the kinetics were comparable to those found for infection with UVPRV. Importantly, neither UVPRV- or LP-mediated escape from silencing depended on PKA or JNK signaling.

US3 is one of two serine-threonine protein kinases encoded by alpha herpesvirus genomes and carried in the virion as an inner tegument protein. The activity of HSV-1 US3 protein kinase has been reported to functionally overlap with PKA [9]. EP0, another tegument component of PRV, is functionally homologous to HSV-1 ICP0, a potent transcriptional activator [10]. Neither the viral PKA analog US3 or the HSV-1 ICP0 homolog EP0 were required to promote escape from PRV silencing, since both UV inactivated EP0 null and Us3 null mutant viruses were able to promote productive infection. Infection with other replication incompetent DNA viruses, such as baculovirus or adenovirus vectors that efficiently transduce neurons could not stimulate escape from silencing.

These results suggest that a generalized cytoplasmic or nuclear response to DNA virus infection is not responsible for the rapid escape from silencing. In our system, efficient reversal of silencing requires the delivery of viral tegument proteins, which must activate multiple cell signaling pathways. Only when viral tegument proteins are not available in the cell bodies (as in the case of reactivation), PRV escape from silencing requires activated host PKA and JNK dependent signaling.

## Results

### The cell bodies that harbor silenced PRV genomes express LAT transcripts

**Fig 1:**
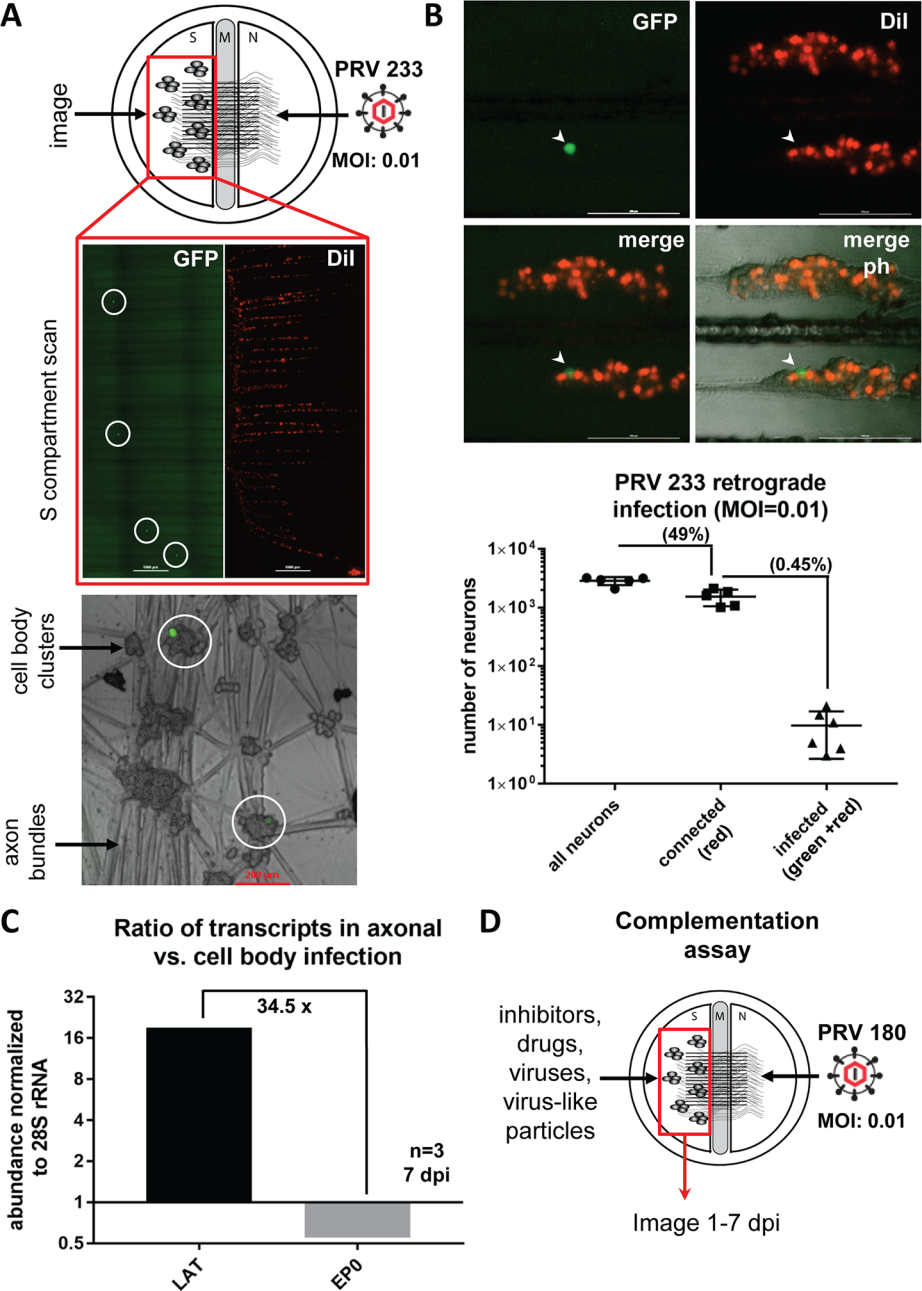
Low MOI axonal PRV infection in compartmented neuronal cultures results in a quiescent infection in a small number of neurons. **(A)** Tri-chamber compartments, S: soma (cell bodies), M: methocel (middle), N: axonal. PRV 233 infection was made in the N compartment at an MOI of 0.01. DiI (red) was added to the N compartment media 6 hpi to label red cell bodies that project axons to the N compartment. Circles highlight primarily infected neurons. Insets show single infected neurons (green) among surrounding non-infected neurons. **(B)** GFP positive, DiI labeled or non-labeled cell bodies were counted at 3 dpi (separate or merged channels are shown, arrowhead points to one GFP positive cell body, ph: phase contrast). Ratios of either red cell bodies to all S compartment neurons (connectivity), or green cell bodies to dual color (green and red) cell bodies (infectivity) were calculated and shown in the graph. **(C)** RNA was isolated after 7 days of either S compartment or N compartment infection with PRV180 at an MOI of 0.01. LAT and EP0 transcripts were quantitated. S compartment (cell body) infection resulted in a productive infection (red capsid accumulation in all of the cells), whereas N compartment (axonal) infection was silent (no detectable red fluorescent signal anywhere in the S compartment). The graph shows the ratio of each transcript in axonal to cell body infection after normalization to 28S rRNA. **(D)** Illustration of the complementation assay: N compartments are infected with PRV180 at an MOI of 0.01 while S compartments are treated with drugs, inhibitors, viruses or virus-like particles.

In our previous report, we established quiescent infections by infecting axons in the axonal “N” compartment of modified Campenot tri-chambers with PRV 180 (which expresses an mRFP-VP26 fusion protein) at an MOI of 0.01 (100 plaque forming units per dish) [5]. At this very low MOI, no cell bodies express any mRFP-VP26 over a period of 3 weeks. Silent genomes can be reactivated after high MOI superinfection of cell bodies with UV treated PRV. This reactivated productive infection then spreads to all neurons in the chamber [5]. These observations raised several important questions including how many cell bodies initially get infected at this very low MOI, and do these silent genomes express latency associated transcripts (LATs), a hallmark of authentic latent infection. To estimate the number of neurons that harbored a quiescent PRV infection, we established quiescent infections using a trans-complemented gB null mutant, which expresses a diffusible GFP under CMV promoter (PRV 233) [11]. This virus is completely deficient in cell-cell spread—only those neurons infected via their axons with PRV 233 (at an MOI of 0.01) expressed the green fluorescent protein in the soma (Fig 1A). We expected the number of cell bodies harboring silent genomes would be small because quiescence was established with approximately 100 infectious particles. We counted on average 9.8 [±2.9 standard error of the mean (SEM)] GFP positive cell bodies in each S compartment after low MOI PRV 233 infection between 3 to 21 days post infection (dpi). We also assessed the number of cell bodies that send axons to the N compartment by adding a lipophilic dye (DiI) in the N compartment. We found that 49% (±4.7% SEM) of neurons were connected to the N compartment, and of these, 0.45% (±0.2% SEM) were infected with PRV 233 (Fig 1B).

Next, we assayed the viral transcripts from the cell bodies either from cultures with silenced PRV 180 genomes (no capsid expression was detected in cell bodies after 7 dpi) or from cultures that were productively infected with PRV 180 by direct infection of cell bodies at the same low MOI (red capsid expression was detected in all cell bodes at 7 dpi). After 7 days, the ratio of LAT to EP0 mRNA was 34.5 fold more in silenced cultures when compared to cultures with productive cell body infection (Fig 1C). In productively infected neurons, the EP0 transcript was 148.3 (±15 SEM) fold more abundant than the LAT transcript. This ratio went down to 4.8 (±0.65 SEM) in quiescently infected neurons at 7 dpi. These experiments showed that PRV infection of axons at an MOI of 0.01 results in a silenced infection in less than 0.5% of connected cell bodies in S compartments. These silenced genomes do not express late genes, indicated by the lack of mRFP-VP26 capsid protein fluorescence, but they do express LAT transcripts, as expected of authentic latent genomes.

### The complementation assay: Activation of PKA by forskolin or dbcAMP in cell bodies results in escape from silencing

We established a system to study the mechanism of how low MOI PRV 180 retrograde infection which is destined to be silenced could be redirected to a productive infection. In these assays, we do not wait 3 weeks for the full establishment of quiescence. Instead, we infect axons with PRV 180 at an MOI of 0.01 as before, but we simultaneously treat or infect cell bodies in S compartments with drugs, inactive/mutant viruses or virus-like particles (Fig 1D). Over 7 days, we monitor mRFP-VP26 expression in the cell bodies to determine the extent of productive PRV 180 infection. In this complementation assay, we call a productive infection an ‘escape from silencing’. This assay is fundamentally distinct from `reactivation assays`that aim to investigate the reversal of repressive genome modifications after latency is established.

**Fig 2:**
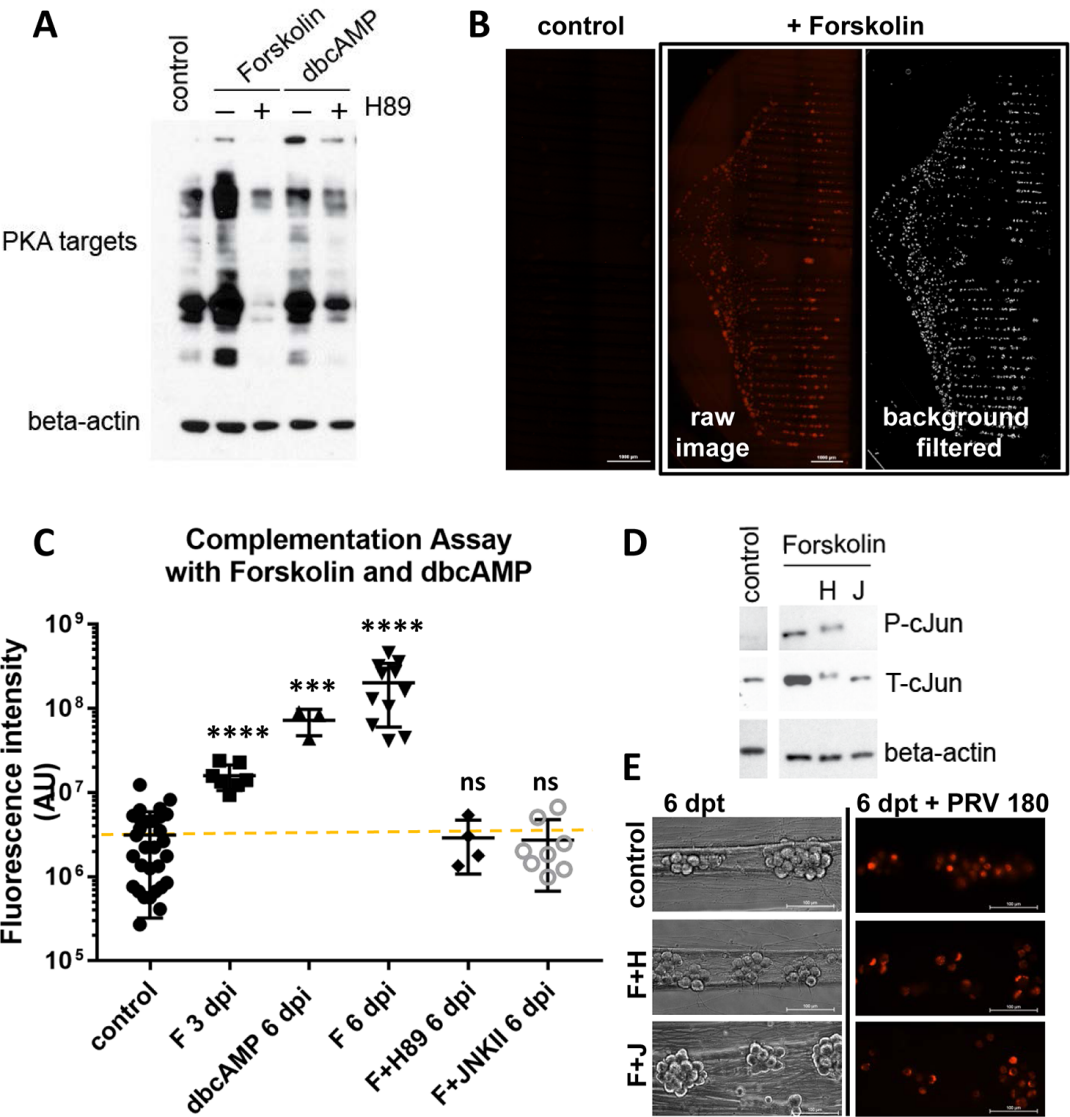
Forskolin and dbcAMP promote escape from silencing in a PKA and JNK dependent manner. **(A)** S compartments were treated either with forskolin or dbcAMP for 3 hours or untreated as a control. The PKA inhibitor H89 was added either at 30 min. post forskolin or dbcAMP treatment (+) or not (-). 3 S compartments were harvested per condition and samples were run on SDS-PAGE. PKA activity was tested using a polyclonal antibody that detected PKA targets. Beta-actin was used as loading control. **(B)** The complementation assay was performed with forskolin treatment and entire S compartment scans for red fluorescence are shown for 1 control and 1 forskolin treated chamber 6 dpi. **(C)** The fluorescence intensity was quantitated for each S compartment scan after background was removed (AU: arbitrary units). Each data point represents one dish (ns is not significant, **** is p<0.0001 and *** is p = 0.0004). **(D)** The efficiency of inhibitors was tested in dissociated neurons in 12 wells (3 wells were pooled per condition). Inhibitors were added 1 h post forskolin treatment. Cells were harvested at 3 hpi (H: H89, J: JNKII). JNK activity was monitored by phospho-c-Jun (P-cJun) and total c-Jun (T-cJun) antibodies. Beta actin was used as loading control. **(E)** Untreated neurons (control) or neurons treated with forskolin plus H89 (F+H) or forskolin plus JNKII (F+J) were imaged at 6 days post treatment (dpt), and then infected with PRV 180 at an MOI of 5. Fluorescent images showing red capsid accumulation in cell bodies were taken 24 hpi.

Because elevated cyclic adenosine monophosphate (cAMP) levels and consequent protein kinase A (PKA) activation have been shown to reactivate quiescent HSV-1 infections [12,13], we first tested the effect of treatments that cause elevated cAMP in our system. Treatments with forskolin or a cell-permeable dibutyryl cyclic AMP (dbcAMP) in the cell body compartment resulted in increased phosphorylation of PKA substrates, and this effect was blocked by additional treatment with a PKA inhibitor, H89 (Fig 2A). To quantitate escape from silencing, we measured the total fluorescence (mRFP-VP26 capsid protein) in chambered neuronal cultures after performing background subtraction and feature selection using Fiji/ImageJ v.1.48u [14,15] (Fig 2B). When applied to cell bodies during low-MOI axonal infection, both forskolin and dbcAMP promoted PRV 180 productive infection (Fig 2C). The mean value of control dishes, where no red capsid protein expression was observed is shown as the baseline. Virus infection spread throughout the S compartment after escape from silencing in approximately 7 days in the case of dbcAMP or forskolin treatment. We further confirmed that the observed effects of forskolin were due to PKA activation by including the PKA inhibitor H89 in the S compartment. Productive infection was blocked when PKA activity was inhibited (Fig 2C), indicating that forskolin-mediated escape from silencing requires PKA activation.

### Forskolin-mediated escape from PRV silencing requires JNK activity

How latent alpha herpesvirus infections reactivate when no viral proteins are produced in the host cell to activate viral transcription is a topic of much research and debate. Recently, Cliffe et al., showed that cJun N-terminal kinase (JNK) activity and c-Jun phosphorylation are essential for HSV-1 reactivation [3]. It was proposed that stress or injury-related stimuli, including phosphatidylinositol 3-kinase (PI3K) inhibition, axotomy, and nerve growth factor (NGF) withdrawal converge on JNK activation and c-Jun phosphorylation for reactivation [3,16]. To understand whether forskolin mediated PRV 180 productive infection (which requires PKA activity) converges on the JNK pathway, we exposed forskolin treated cell bodies to 20 μM of the JNK inhibitor, JNKII, while simultaneously infecting their axons with low MOI PRV 180. In this condition, inhibiting JNK activity prevented the onset of PRV 180 productive infection (Fig 2C). The effect of this inhibitor was confirmed by monitoring the levels of total c-Jun (T-cJun) and phospho c-Jun (P-cJun). JNKII substantially reduced forskolin-stimulated c-Jun accumulation and completely blocked cJun activation (Fig 2D). We also checked c-Jun levels when the PKA inhibitor H89 was included in forskolin treated samples. We detected less c-Jun accumulation, but phosphorylation was not blocked. Also, there was a clear shift in the molecular weight of both total and activated forms of c-Jun. To confirm that chemical inhibitor treatment did not alter viability or virus production capacity of neurons, we monitored neuronal morphology and virus infection under conditions that did not allow PRV productive infection (forskolin+H89 and forskolin+JNKII). After 6 days, forskolin+H89 or forskolin+JNKII treated neurons displayed comparable morphology to untreated neurons, and PRV 180 infection, at an MOI of 5, proceeded comparably in all conditions (Fig 2E). Thus, when no viral components are delivered, PRV 180 escape from silencing occurs via a slow, PKA-dependent activation of the JNK pathway.

### Infection of cell bodies with UV treated PRV stimulates rapid escape from silencing

**Fig 3:**
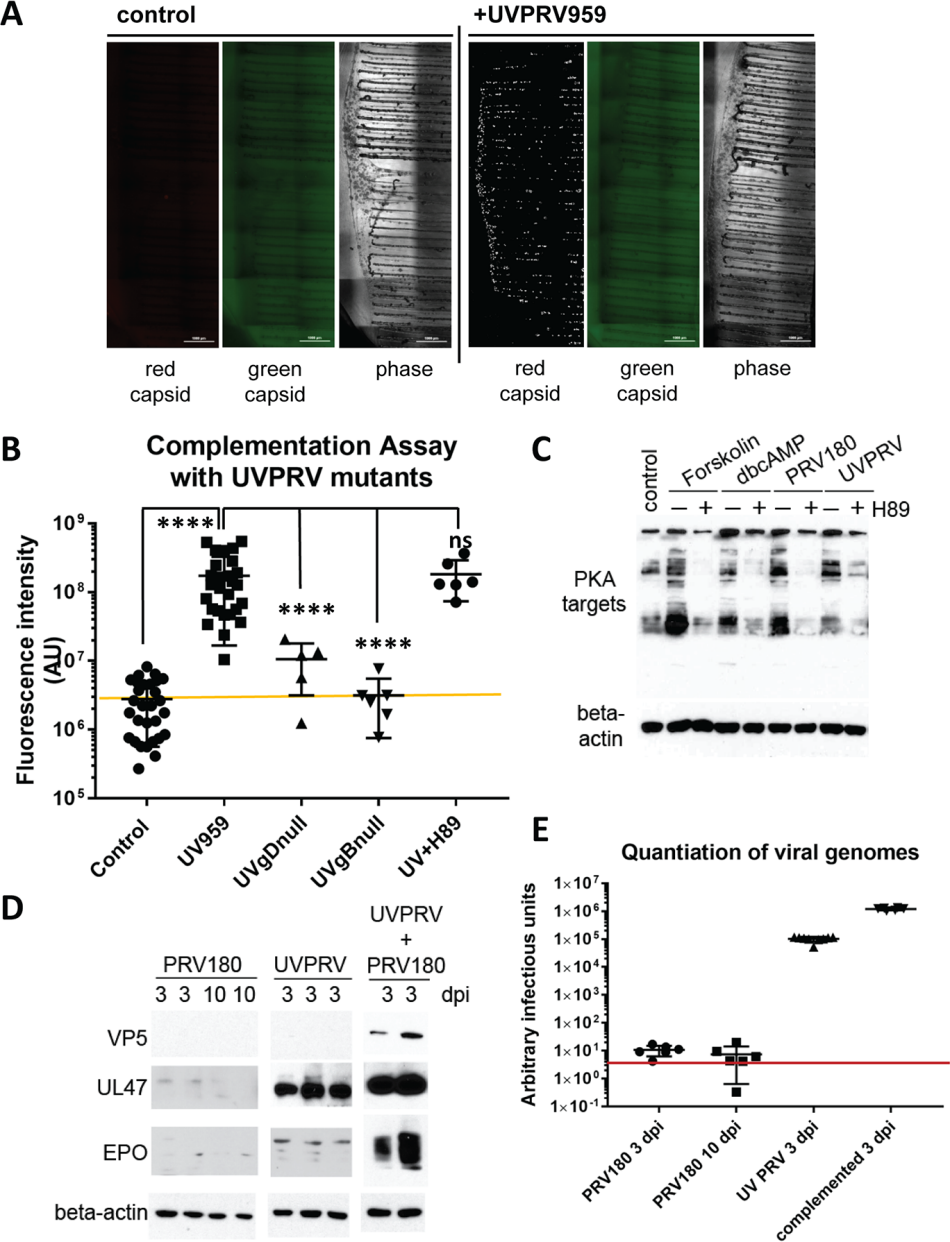
Simultaneous infection of cell bodies with UVPRV enables axonally infecting PRV180 to escape from silencing. **(A)** Control and UVPRV959 (MOI 10) complemented S compartments are shown (3 dpi). Red channel is shown after background filtration. Raw image is used for the green channel (no signal). **(B)** Quantitation of imaging data was graphed including control dishes (7 dpi), UV959 (3 dpi), UVgBnull or UVgDnull PRV complementation (7 dpi), and UV959 plus H89 (UVH89) complementation (3 dpi). Each data point represents one dish (ns is not significant and **** is p<0.0001). **(C)** Western blot analysis of PKA targets after 3 hours of PRV180 and UVPRV infection in S compartments at an MOI of 10 with (+) or without (-) H89 treatment are shown in comparison to forskolin and dbcAMP treatments. **(D)** Control dishes with axonal PRV180 infection (MOI 0.01) were harvested either 3 or 10 dpi. S compartments infected only with UVPRV (MOI 10), and complementation assay samples were harvested at 3 dpi. The presence of viral capsid (VP5) and tegument (UL47 and EP0) proteins was determined by WB using half of the lysates (each lane represents contents of one S compartment). Beta-actin was used as a loading control. The other half of the lysates was used to quantitate the amount of viral DNA. **(E)** PRV DNA was quantitated using UL54 primers. DNA amounts calculated based on threshold cycle values are shown in the graph. Red line shows the detection limit.

We showed previously that superinfection of cell bodies with UV treated PRV is sufficient to reactivate quiescent PRV genomes [5]. Using our complementation assay, we asked whether virion components delivered to cell bodies can trigger escape from silencing similar to that observed with forskolin treatment. We infected cell bodies in S compartments with a high MOI of UV inactivated PRV 959 (mNeonGreen-VP26, a green fusion protein; UVPRV) [5], while simultaneously infecting axons with PRV 180 at low MOI. We chose to use green capsid PRV to be able to monitor the effect of UV inactivation. UV inactivated virus particles, as we have previously reported, are able to enter, undergo retrograde axonal transport in neurons, and deliver their defective genomes to the nuclei [5]. UVPRV does not exclude superinfection with a second PRV genome [17]. Simultaneous co-infection of cell bodies with high MOI UVPRV enabled efficient escape from silencing by PRV 180, as indicated by red capsid signal in the cell bodies (Fig 3A). Note that there is no green capsid signal in the cell bodies, confirming that the UV inactivated virus is unable to replicate or express late viral genes. Surprisingly, PRV 180 infection spread to all neurons in the S compartment in only 3 days following addition of UVPRV to the cell bodies. This is considerably faster than the 7 day period required for PKA-induced escape from silencing. Entry of UVPRV was required because escape from silencing was not observed with UV inactivated non-complemented entry deficient mutants (UVgBnull or UVgDnull) (Fig 3B). Interestingly, while the PKA inhibitor H89 efficiently blocked the activity of host PKA (Fig 3C), H89 had no effect on UVPRV-mediated escape from silencing (Fig 3B). We also confirmed that PRV 180 or UVPRV infection of cell bodies induces PKA activity early after infection (3 hpi), and this activity is also efficiently inhibited by the addition of H89 1 hpi (Fig 3C). These observations suggest that, upon cell body entry, components of UV treated virions trigger an accelerated escape from silencing through PKA-independent mechanisms.

We also monitored viral DNA replication and expression levels of the major capsid protein (VP5), the most abundant tegument protein (UL47) [18], and the lytic transactivator protein (EP0) in neuronal cultures infected by either PRV 180 alone in N compartments (3 day or 10 day post infection-dpi), UVPRV alone in S compartments (3 dpi), or both of them simultaneously as we did in the complementation assay (3 dpi). We were able to detect the major capsid protein VP5 only when PRV 180 escaped from silencing in the complementation assay (Fig 3D). Tegument protein UL47 was detectable in cell bodies infected with UVPRV but the levels were less than in the co-infected samples. We detected EP0 in UVPRV infected cell bodies, but much less than UL47 in the same samples. EP0 reached high levels during PRV 180 productive infection stimulated by UVPRV complementation (Fig 3D). We quantitated viral DNA amounts in cell bodies in these three conditions. In a silent infection (PRV 180 alone), DNA amounts were almost at the detection limit of our Q-PCR system (CT_mean_ = 33.76 ± 0.29 SEM at 3 dpi, CT_mean_ = 34.91 ± 0.08 SEM at 10 dpi). UVPRV infection of cell bodies yielded approximately 8x10^3^ fold more DNA than PRV 180 alone (CT_mean_ = 20.44 ± 0.12 SEM), and approximately 12.8 fold less DNA than the complemented samples (CT_mean_ = 16.76 ± 0.06 SEM) (Fig 3E). These results show that UV treated PRV particles deliver significant amounts of tegument proteins as well as replication deficient viral DNA. Clearly one or both of these components trigger escape from silencing in the complementation assay.

### Light particles promote PRV 180 escape from silencing

**Fig 4:**
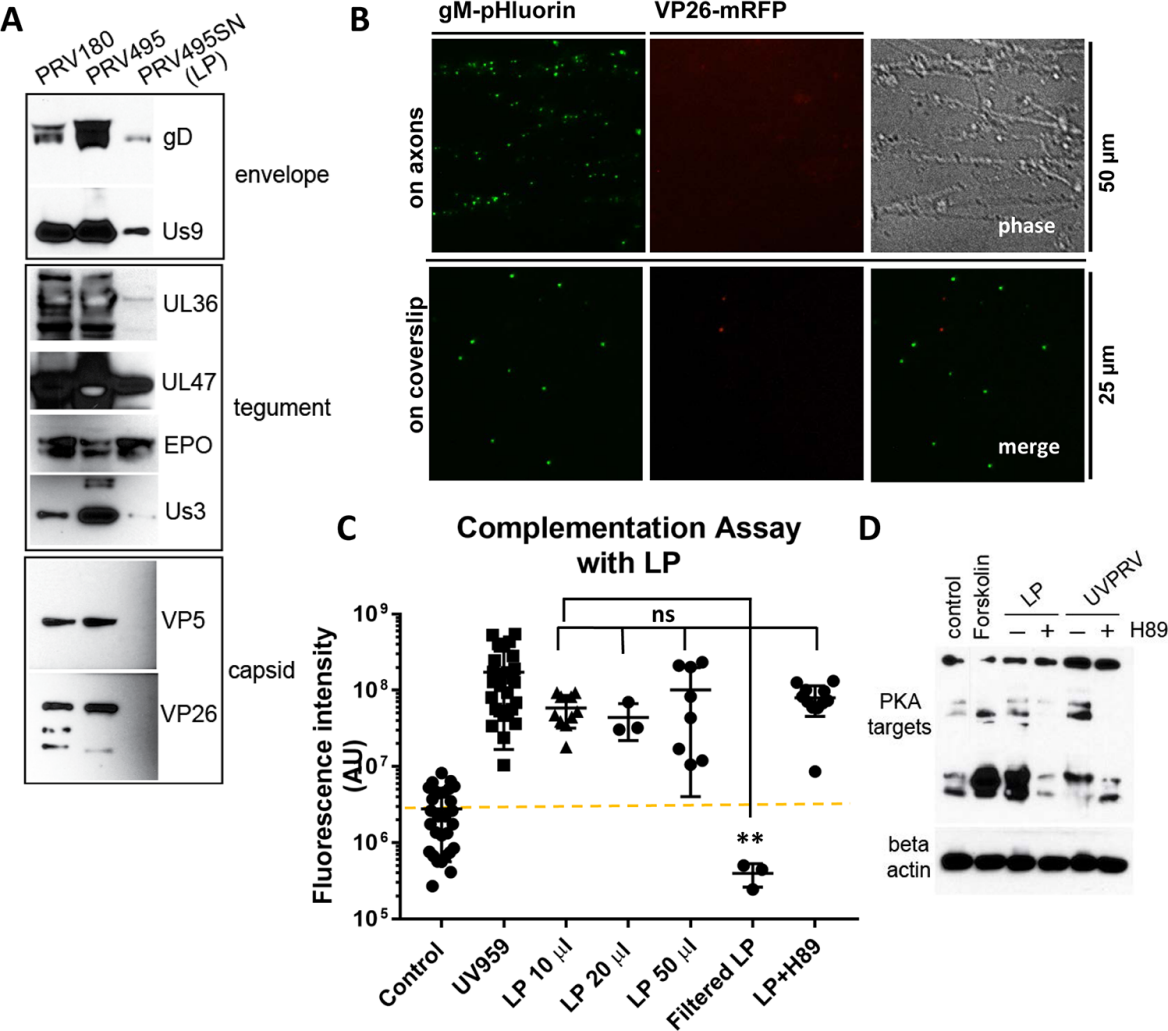
LP promote escape from PRV180 silencing independent of PKA activity. **(A)** 20 μl of PRV 180, complemented PRV 495 (produced after infecting UL25 expressing PK15 cells) stocks, and light particles (LP) prepared from PRV 495 infected PK15 supernatant (SN) were fractionated by SDS-PAGE. The presence and quantity of envelope (gD and Us9), tegument (UL36, UL47, EP0, and Us3), and capsid (VP5 and VP26) proteins were determined using specific antibodies (first two lanes were overexposed to be able to detect the proteins in the LP lane). **(B)** 50 μl LP were added to axons in the N compartment, and gM-pHluorin positive particles were visualized (no red particles were detected). The same amount of LP was also put on a glass coverslip and screened for green, red or dual color particles. **(C)** Complementation assays were performed using different amounts of LP with or without H89 (3 dpi). Each data point represents one dish (ns is not significant and ** is p = 0.0035). **(D)** Western blot analysis of PKA targets was done after 3 hrs of UVPRV or LP infection with (+) or without (-) H89 treatment. Beta-actin was used as a loading control.

We hypothesized that PRV quiescence is established after low MOI axonal infection, either due to insufficient tegument proteins delivered to the nuclei to transactivate viral gene expression, or insufficient genomes to saturate the silencing machinery. We were able to test these ideas using light particles (LP). We used cell supernatants from PRV UL25 null mutant infected cells. The UL25 protein is essential for proper genome encapsidation, capsid nuclear egress, and production of infectious progeny virions [19]. This mutant produces almost no infectious virions, but produces abundant virion-like light particles (LP) that contain tegument but do not contain capsids or genomes. We constructed PRV 495, a double recombinant that expresses two fluorescent fusion proteins: gM-pHluorin, a green fluorescent viral glycoprotein that is incorporated into light particles, and mRFP-VP26 (red capsid) [7,20]. The PRV 495 recombinant was prepared by infecting UL25-expressing helper PK15 cells. We prepared a capsid-free LP stock by infecting non-complementing PK15 cells with complemented PRV 495 at an MOI of 5. Varying amounts of this LP stock were run on SDS gels, and we assessed the presence of envelope proteins (gD, Us9), outer tegument (UL47 and EP0) and inner tegument proteins (UL36, Us3) (Fig 4A). Inner tegument proteins UL36 and Us3 were much less than the other tegument components UL47 and EP0. The lack of capsid proteins (VP26 and VP5) was confirmed by WB and by fluorescence microscopy of LP inoculum on axons and adhered to glass coverslips (Fig 4A and 4B). Many gM-pHluorin positive punctae were detected attached to axons at 1 h post-inoculation. We did not detect any dual color puncta on axons or coverslips, confirming that the PRV 495 LP preparation does not contain viral capsids. We noticed some rare single color red punctae that did not colocalize with the green gM signal, which possibly represents fluorescent debris from lysed PRV 495-infected cells (Fig 4B). We then inoculated cell bodies with the LP preparation to determine if LP could promote escape from PRV 180 silencing. PRV 495 LP promoted escape from silencing as fast as the UVPRV (Fig 4C). This effect was not dose dependent using the amounts we tested (10, 20 and 50 μl). Since there were no detectable capsids in our LP preparations, we concluded that viral genomes were not necessary for escape from silencing. To be sure that the LP-mediated escape from silencing is due to LP and not due to other soluble factors released from infected cells, we filtered the LP preparation through a 0.1 μm filter. This filtered LP preparation did not promote escape from silencing (Fig 4C). This indicates that the “escape from silencing” activity is particulate, and secreted viral/host proteins or smaller cellular exosomes are not able to promote escape from silencing. Moreover, similar to what was observed with UVPRV, the PKA inhibitor H89 was not able to block LP-induced escape from silencing (Fig 4C). LP infection of cell bodies induced phosphorylation of PKA targets comparable to what we found with UVPRV, and this induction was blocked by the addition of H89 (Fig 4D). These data confirmed that in the absence of capsids or genomes, viral proteins incorporated into particles are sufficient to induce an accelerated escape from silencing within 3 days, and this escape does not require PKA activity.

### PRV tegument proteins Us3 and EP0 are not required for escape from silencing

**Fig 5:**
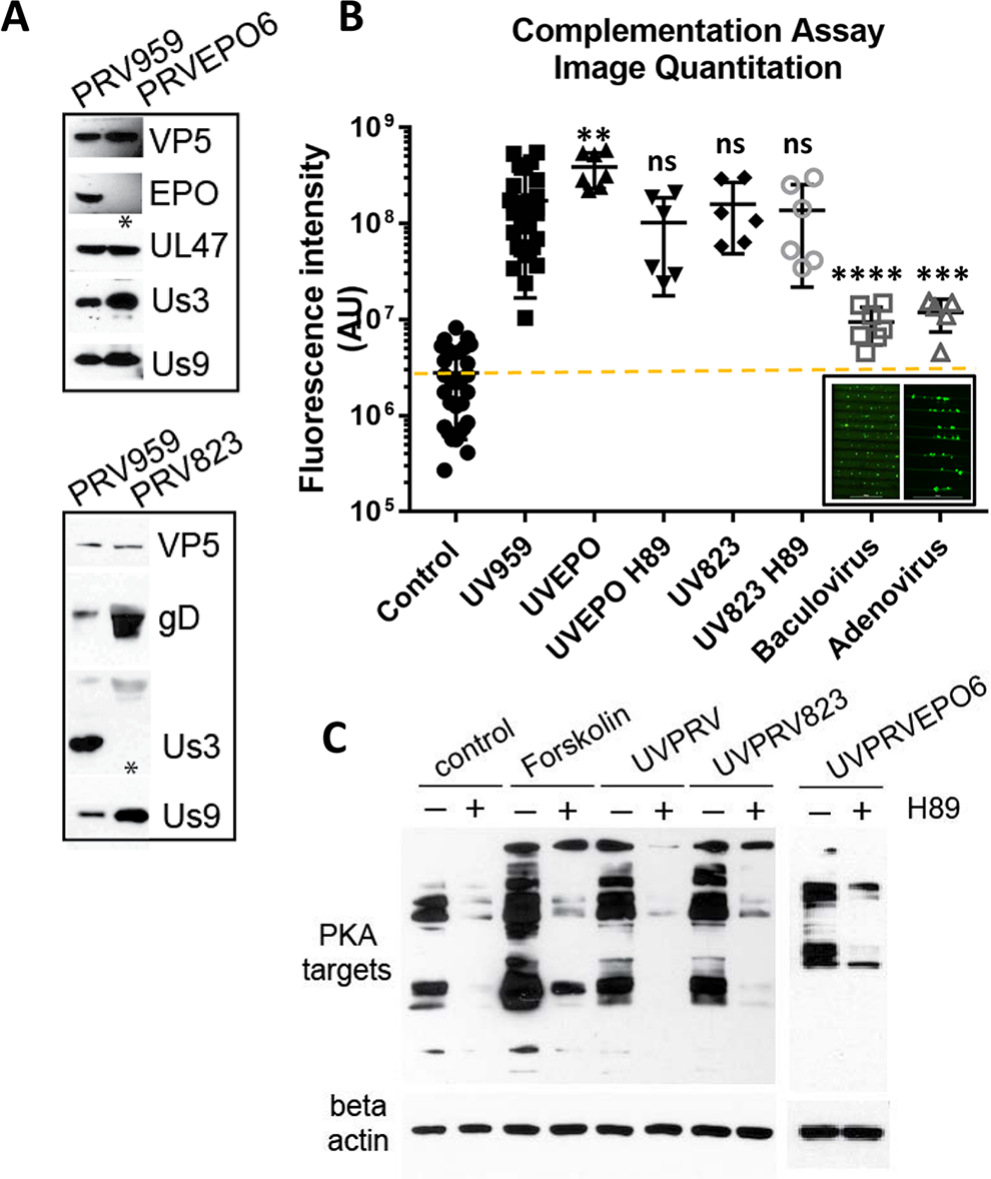
PRV tegument proteins, US3 and EP0 are not required to promote PRV180 escape from silencing. **(A)** 20 μl PRV EP0null (PRVEP06) and Us3null (PRV 823) virus stocks were run on SDS-PAGE in comparison with PRV959 stocks. **(B)** Complementation assays were performed using UV inactivated PRV EP06 (UVEP06) and PRV 823 (UV823) with or without H89 (3 dpi). Each data point represents one dish (ns is not significant, **** is p<0.0001, *** is p = 0.0004 and ** is p = 0.0021). Replication incompetent DNA viruses expressing GFP (baculovirus and adenovirus) were also included. Inset shows the transduction efficiencies of baculovirus and adenovirus in S compartments. **(C)** Western blot analysis of PKA targets during 3 hrs of UVPRV (PRV 959), UV823 or UVEP06 infection with (+) or without (-) H89 treatment. The samples are compared to control and forskolin treatment. Beta-actin was used as a loading control.

We hypothesized that a tegument protein delivered by UVPRV or LP might trigger escape from silencing when PKA signaling is blocked. Specifically, we looked at PRV Us3, a potential analog of PKA [9], and PRV EP0, a functional homolog of HSV-1 ICP0, which contains a putative cAMP response element in its promoter [10]. To understand whether the PRV tegument components Us3 or EP0 play a major role in escape from silencing, we prepared stocks of viral mutants that do not express these proteins (Fig 5A). PRV 823 [21] contains a Us3 null mutation, and PRV EPO6 contains an EP0 null mutation (see Materials and Methods). We then performed the complementation assay by infecting cell bodies in the S compartment with UV inactivated EP0 null (UVEPO6) or Us3null (UV823) while simultaneously infecting axons with low MOI PRV180 (Fig 5B). The UV inactivated EPO null mutant promoted escape from silencing with comparable kinetics to UVPRV and LP in a PKA independent manner. Unexpectedly, we also found that the Us3null mutant promoted escape from silencing with or without H89 treatment (Fig 5B). Infection of neurons with either of these mutants induced PKA activity in 3 hours comparable to UVPRV and H89 blocked this activity when added 1 hour after infection with either PRV mutant (Fig 5C).

These data suggest that UVPRV- and LP-mediated escape from silencing involves multiple signaling pathways, which may be distinct from the forskolin- and dbcAMP-mediated PKA pathway that induces a slower escape from silencing. Alternatively, another viral kinase in the tegument, which is resistant to H89, may be able to activate productive infection by phosphorylating PKA targets (e.g. UL13).

We entertained the hypothesis that UVPRV and LP infections stimulated a general cell response to virus infection, which resulted in escape from silencing. We tested replication deficient adenovirus and baculovirus recombinants expressing diffusible GFP to determine if high MOI infection of cell bodies promoted PRV 180 escape from silencing. Although both DNA virions (one naked, one enveloped) efficiently transduced GFP expression in neuronal cell bodies, neither promoted PRV 180 escape from silencing (Fig 5B). We conclude that escape from silencing by superinfection with UVPRV or LP is not a generalized neuronal response to DNA virus infection. Rather, it is a herpesvirus-specific induction of productive cycle viral promoters by multiple tegument-host protein interactions or induction of injury response pathways.

### UV inactivated PRV- and LP-mediated escape from silencing does not depend on JNK activity

**Fig 6:**
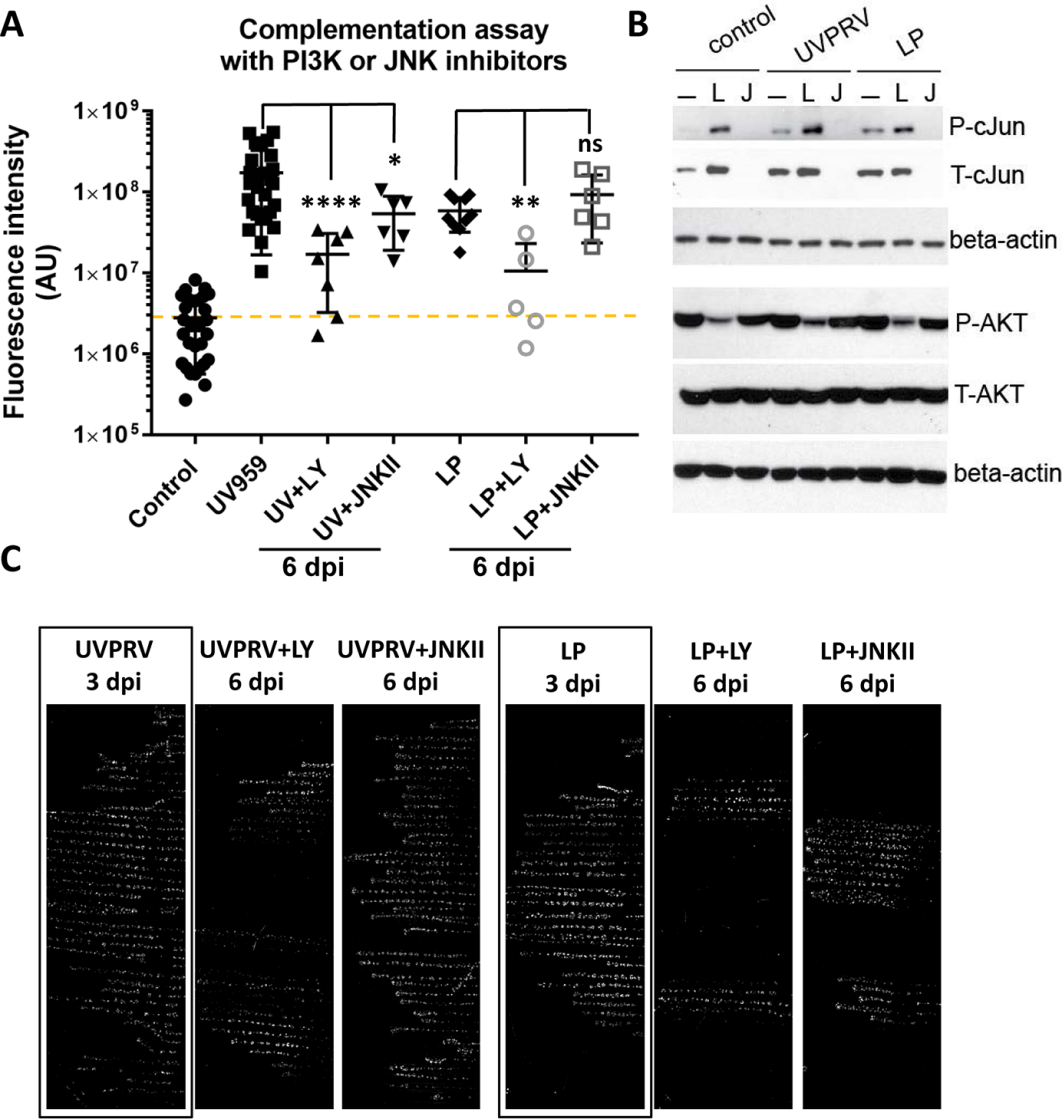
JNK inhibition does not block UVPRV or LP promoted escape from silencing. **(A)** Complementation assays were performed using UVPRV and LP with or without the PI3K inhibitor LY294002, or the JNK inhibitor JNKII (added 1 hpi) (6 dpi). Each data point represents one dish (ns is not significant, **** is p<0.0001, ** is p = 0.0013 and * is p = 0.033). **(B)** The efficiency of inhibitors was tested in dissociated neurons in 12 wells (3 wells were pooled per condition). The inhibitors were added 1 h post UVPRV or LP infection. Cells were harvested 3 hpi (L: LY294002, J: JNKII). JNK activity was monitored by phospho-c-Jun (P-cJun) and total c-Jun (T-cJun) antibodies. PI3K activity was monitored by comparing steady state levels of phospho-AKT (P-AKT). Beta actin was used as a loading control. **(C)** Representative S compartment scans (background removed) are shown for conditions: UVPRV plus LY/JNKII or LP plus LY/JNKII (6 dpi) in comparison to UVPRV or LP mediated complementation at 3 dpi.

Our findings that UVPRV and LP mediate an accelerated, PKA-independent escape from silencing support the hypothesis that the molecular mechanism(s) promoting quiescence or productive infection might differ from the mechanisms of de-repressing viral promoters during reactivation from latency. To test whether escape from silencing mediated by UVPRV or LP is regulated by JNK activity, we infected axons with PRV 180, simultaneously treated cell bodies with UVPRV or LP, and subsequently added a JNK inhibitor (JNKII) one hour post infection. When JNK activity was inhibited, neither UVPRV- nor LP-mediated escape from silencing was blocked, but the spread of infection among the S compartment neurons was delayed (Fig 6A and 6C). We observed red capsid accumulation starting at 3 dpi, but the full spread of infection took 6 days (Fig 6C). We also treated UVPRV or LP infected cell bodies with the PI3K inhibitor, LY294002. In this case, the onset and spread of PRV 180 productive infection was significantly delayed but not completely blocked (Fig 6A and 6C). Interestingly, we saw “strings” of infected neurons in S compartments (Fig 6C showing limited spread of infection), but full spread of infection was prevented particularly when PI3K activity was inhibited. These findings are consistent with the literature indicating a role for this kinase in herpesvirus infection (see [22] for review). The activity of the inhibitors also was tested in mock, UVPRV and LP treated neurons. Interestingly, treatment of neurons with UVPRV or LP increased total and phosphorylated c-Jun levels comparable to forskolin. JNKII blocked c-Jun phosphorylation in all of these samples (Fig 6B), but it interfered only with forskolin-mediated and not with UVPRV- or LP-induced escape from silencing. LY294002 effectively reduced activated AKT levels (pAKT) as expected, and induced c-Jun accumulation and phosphorylation in all of the conditions where the inhibitor was added (Fig 6B). These results further established that UVPRV- and LP-mediated escape from silencing proceeds through a distinct molecular mechanism from cAMP/PKA and JNK-mediated activation of productive infection.

## Discussion

Alpha herpesviruses establish latency in peripheral nervous system (PNS) ganglia *in vivo*. However, *in vitro* HSV-1 or PRV infections of dissociated PNS neurons in culture (e.g. trigeminal ganglia, dorsal root ganglia, or superior cervical ganglia) generally result in productive infection unless viral DNA replication is suppressed using DNA synthesis inhibitors or interferon treatment [23]. Using our *in vitro* model system, which mimics the natural route of neuronal invasion by long distance retrograde axonal transport, we previously showed that PRV infection of axons at a low MOI (below 0.1) results in reactivateable quiescent infections in distant cell bodies. Importantly, this was the first *in vitro* system for establishing PRV quiescence without the use of DNA synthesis inhibitors or cytokine treatment. These findings raised the question of why the axonally delivered viral genomes can reach the cell bodies but cannot initiate a productive infection. In the current study, we aimed to uncover cellular and viral components that prevent establishment of quiescent infection and promote productive replication of axonally delivered genomes (i.e. escape from silencing) after low MOI infection of axons.

We developed a compartmented complementation assay where axons are infected with a PRV recombinant that expresses mRFP-VP26 (red capsid virus) at MOI of 0.01, which produces a silenced, quiescent infection. Cell bodies in a separate compartment from axons are simultaneously treated with different reagents, UV inactivated virus or virus-like particles. Using this system, we reveal two distinct molecular mechanisms to start productive infection from quiescently destined PRV genomes: host stress-mediated (slow) and viral tegument-mediated (fast). Host stress mediated productive infection required both PKA and JNK activity, whereas viral tegument mediated productive infection did not. When cell bodies were treated with forskolin or a cell permeable cAMP analogue, PKA was activated. This resulted in escape from silencing and productive infection spreading to all cell bodies in the S compartment in approximately 7 days. We also showed that productive infection was completely lost when PKA activity was blocked. Consistent with the literature, such escape from silencing required stress-activated protein kinase (e.g. JNK) activity [3,16]. However, when S compartment neurons were complemented with a UV inactivated PRV, the axonal PRV 180 infection (which would otherwise be destined for quiescence) escaped silencing rapidly and spread to all neurons in the S compartment in 3 days. Infection by entry deficient UVPRV gBnull or gDnull mutants did not induce escape from silencing showing that particles must fuse and release viral components into the neuronal cell body to stimulate a productive infection. Moreover, escape from silencing did not require tegument proteins Us3 or EP0, and surprisingly did not depend on cellular PKA or JNK activity.

From the large body of literature surrounding alpha herpesvirus latency, several hypotheses emerge to explain why these viruses stay quiescent in PNS neurons *in vivo*. One hypothesis is that separate long-distance axonal transport of nucleocapsids and certain tegument proteins (e.g. VP16) challenges the timely arrival of tegument and viral DNA in the nucleus [24]. If these components do not reach the nucleus concurrently, VP16 (along with cellular proteins (HCF1, oct1, LSD1)) may not be able to orchestrate the transactivation of immediate early genes (e.g. ICP0) [25]. Furthermore, upon the nuclear delivery of the herpesvirus genome, ND10 (nuclear domain 10) proteins assemble at the viral DNA and viral genomes are covered with repressive histones [26] (see [27] for review). The newly made ICP0 protein removes repressive histone modifications to activate early/late gene expression [27–30].

A second idea is that productive infection can initiate only after sufficient genomes are delivered to the neuronal nuclei to saturate the silencing complexes (e.g. ND10, Co-REST, HDACs). Because only a limited number of alpha herpesvirus genomes can be expressed (fewer than 7) in a given neuronal or epithelial cell even after high MOI infection (100 pfu/cell), the remaining genomes might be targeted by the silencing complexes, resulting in quiescent infection [31].

To test the hypothesis that alpha herpesvirus tegument proteins alone are sufficient to trigger escape from silencing, we complemented S compartment cell bodies with light particles. PRV light particles without capsids were produced during infection by UL25 null PRV mutant. These light particles were able to initiate productive infection of axonally delivered PRV 180 as fast as UVPRV and did so in a PKA- and JNK-independent manner. Although herpesvirus infected cells yield large amounts of capsid-less virus-like particles (up to 81% in PRV [7], and 84% in EBV infection [8]) that contain viral envelope and tegument proteins and use exocytosis machinery similar to infectious virions [6–8,32], their function is not well understood. Recently, Gong et al., showed that such virus-like particles produced by Kaposi`s sarcoma associated virus (KSHV) induce B-cell differentiation signaling to promote lytic infection suggesting a role for light particles in productive versus latency switch [8].

Our *in vitro* findings support the first hypothesis: Low MOI axonal infection is destined for quiescence most likely due to a lack of tegument proteins in the neuronal cell body and nucleus, which are required to initiate viral gene transcription and productive infection. This conclusion is based on results from the PRV light particle complementation assays, which suggest that excess genomes do not play a major role in escape from silencing. However, viral tegument proteins are critical for redirecting low MOI axonal infection from quiescent to productive. We have yet to identify the specific tegument or tegument proteins responsible, but potential candidates include VP16 and UL13 kinase. It is possible that multiple components are required, and a single viral protein may not be sufficient to prevent quiescence.

A remaining question concerns how PKA activation leads to JNK mediated c-Jun phosphorylation. One idea is that cAMP-mediated PKA activity leads to direct activation of the dual leucine zipper kinase DLK that is upstream of JNK. This pathway has recently been shown to mediate axonal regeneration [33] and also HSV-1 reactivation [3]. Another possibility is that elevated cAMP levels activate PKA, which in turn phosphorylates mitogen activated protein kinase 4 (MKK4). MKK4 also directly phosphorylates and activates JNK (see [34] for review), and MKK4 has been shown to mediate aromatase expression in response to prostaglandin E_2_ in a cAMP/MKK/JNK dependent manner [35].

Our low MOI axonal infection model of silencing suggests that very few cell bodies harbor silenced genomes after axonal infection, and this raises the question of how this compares to *in vivo* latency models. Using a spread deficient PRV (gBnull) mutant, we found the number of cell bodies in the S compartment with quiescent PRV infections was 9.8 on average. This is approximately 0.5% of the neurons that send axons to the N compartment. Even from this small number of cells, we detected a significant increase in expression of the PRV latency associated transcript in cultures with silenced PRV 180 infection compared to a productive infection. Because it is a hallmark of authentic latent infection, increased LAT expression validates our *in vitro* model of quiescence.

The *in vivo* ocular HSV-1 latency model shows that thousands of neurons in the trigeminal ganglia harbor latent infections, and some of them undergo productive infection before genomes are silenced. We do not see this in our system because we do not see late gene expression as monitored by mRFP-VP26 fluorescent protein expression. More interestingly, in the ocular infection model, only 0.04% of the latently infected neurons reactivate (2–4 neurons/ganglion) and extremely low level replication was detected in these neurons [36–38]. Unlike this small percentage of reactivation in the animal models, we see extensive spread rather than low level viral replication after productive infection is initiated. This difference is most probably due to the lack of immune system control in our simplified *in vitro* model; as soon as productive infection starts it spreads to all neurons unless replication inhibitors or cytokines are added.

A two-stage program initiated by a pre-existing epigenetic switch has been proposed for HSV-1 reactivation from latency [3,16]. The program includes a generalized de-repression of viral promoters (phase I or animation) leading to *de novo* synthesis of many viral proteins. In the first phase, viral gene expression does not follow the regular early-to-late gene-cascade, and is not dependent on VP16 activity. *Ex vivo* reactivation triggered by axotomy in combination with neurotropin deprivation follows such a disordered pattern of gene expression. The second phase of reactivation closely resembles productive infection: It follows the expected transcription cascade and chromatin remodeling that is critical for full reactivation [16]. More recently, Sawtell and Thompson proposed a three-step program including pre-initiation, initiation, and progression [39]. Interestingly, explant reactivation produces new virus much faster than reactivation through PI3K-inhibition [40]. It may be that, multiple signaling pathways were activated during dissection of the ganglia that could accelerate the proposed biphasic program. UVPRV- or LP-mediated escape from silencing shows a similar rapid pattern: If viral tegument proteins are delivered into the cell body, several signaling pathways are induced, and neither PKA nor JNK seem to be a major player. In this case, productive infection proceeds rapidly. If tegument proteins are not efficiently delivered to the cell body (as in the case of axonal entry and similar to reactivation), PKA and JNK activity may help de-repression or transcription of incoming viral genomes. This process takes a longer time similar to PI3K inhibition-mediated HSV-1 reactivation.

We still do not know if escape from silencing differs mechanistically from reactivation from latency, or whether forskolin-induced productive infection observed in our system is more akin to reactivation rather than escape from quiescence. Our compartmented complementation assay will help dissect these phenomena by investigating whether viral lytic promoters are associated with histone H3 tri-methylation at lysine 27 (H3K27me3) as shown for the HSV-1 genome [41,42]. Future studies will also explore the effect of type I and II interferon, as well as stress hormones (epinephrine and corticosteroids) on the establishment of silent PRV infection as well as escape from genome silencing.

## Materials and methods

### Cell lines and viruses

Porcine kidney epithelial cells (PK15; ATCC) were used to produce and titer PRV. These cells were maintained in Dulbecco’s modified Eagle medium (DMEM) supplemented with 10% fetal bovine serum, 1% penicillin and streptomycin. PRV Becker is a wild-type laboratory strain [43]. PRV 180 expresses an mRFP-VP26 fusion protein in a PRV-Becker background [44]. PRV 959 expresses an mNeonGreen-VP26 fusion protein in a PRV Becker background [5]. PRV 233 is a gB-null mutant of PRV-Becker expressing diffusible GFP from the gG locus under the control of a cytomegalovirus (CMV) promoter [11]. PRV 233 stocks were propagated, and their titers were determined in PK15 cells stably transfected with LP64e3, a plasmid expressing PRV gB (constructed by Lisa Pomeranz in the Enquist lab). PRV 357 is a gD-null recombinant expressing diffusible GFP in a PRV Becker background [45]. PRV 357 is grown G5 cells that constitutively express PRV gD [46]. To obtain entry deficient virus stocks, non-complementing PK15 cells were infected with PRV 233 and PRV 357 at high MOI, and harvested at 24 hpi to obtain gB null and gD null virions, respectively. The titers of these stocks were determined based on the genomic DNA content by quantitative RT-PCR. Mutant stock titers were normalized using volumes of each stock corresponding to the amount of DNA in 10^6^ pfu of *wt* PRV. PRV 823 is Us3 null of PRV-Becker expressing mRFP-VP26 fusion protein [21] and PRV EP06 is EP0null expressing mNeonGreen-VP26 in a PRV-Becker background (constructed by Hao Oliver Huang in the Enquist lab). PRV 495 is UL25-null expressing mRFP-VP26 and gM-pHluorin fusion proteins [20]. Complementing cell line expressing PRV UL25 protein was a kind gift from G. Smith, Northwestern University [47].

### Primary neuron cultures

Superior cervical ganglia (SCGs) were isolated from embryonic day 17 Sprague-Dawley rat embryos (Hilltop Labs). Primary neurons were cultured in tri-chamber dishes as previously described [48,49]. Multi-well or 35-mm plastic tissue culture dishes (Falcon) or optical plastic dishes (Ibidi) were coated with 500 μg/ml of poly-DL-ornithine (Sigma Aldrich) and 10 μg/ml of natural mouse laminin (Invitrogen). After coating, two sets of grooves were etched on the dishes. A silicone grease-coated tri-chamber (Tyler Research) was placed on top of a drop of 1% methylcellulose (in neuronal medium) covering the groves. Ganglia were trypsinized and triturated, and approximately 2/3 of an SCG was plated in the S compartment. Neurons were maintained in complete neuronal medium: Neurobasal medium (Gibco) supplemented with 100 ng/ml nerve growth factor 2.5S (Invitrogen), 2% B27 (Gibco) and 1% penicillin and streptomycin with 2 mM glutamine (Invitrogen). 2 to 3 days after seeding, 1 mM cytosine-D-arabinofuranoside (AraC; Sigma-Aldrich) was added for at least 2 days to eliminate non-neuronal cells. Neurons were cultured for 14–21 days prior to experiments.

### Virus infection and drug treatment in compartmented neuronal cultures

Before adding the virus inoculum to N and/or S compartments in tri-chambers, 1% methylcellulose prepared in neuronal medium was added in the M (Middle) compartment to prevent any possible leakage from either of these compartments. Neuronal infections were done using the indicated amounts of recombinant PRV. DiI was added at 2.5 μg/mL (Life Technologies) to the N compartment. Reagents used in this study: Forskolin (Sigma), dibutyryl cAMP (Selleckchem), H89 (Selleckchem), LY294002 (Sigma) and JNK inhibitor II (Calbiochem).

### Microscopy

Imaging was performed on a previously described Nikon Ti-E inverted epifluorescence microscope [50]. Tiled images of the entire S compartment were captured using Nikon NIS Elements software, a Cool Snap ES2 camera (Photometrics), and 4x magnification objective. To measure the total fluorescence in chambered neuronal cultures, we performed background subtraction and feature selection using Fiji/ImageJ v.1.48u [14]. Because the background fluorescence intensity is highly variable across tiled images, we first subtracted the local background by calculating a 20 pixel radius median filter and subtracting this median filtered image from the original. To select fluorescent cell bodies, we next performed granulometric filtering using the GranFilter plugin in Fiji/ImageJ [15]. GranFilter plugin settings were as follows: circular structure element, 3 pixel radius, 6 pixel step size. With these settings GranFilter selects fluorescent structures that are the approximate size and shape of fluorescent neuronal cell bodies. We then manually set a region of interest to exclude image artifacts caused by reflections off the chamber walls and grooves. Finally, we measured the integrated fluorescence intensity using the Measure function in Fiji/ImageJ and exported these measurements to Microsoft Excel. High magnification imaging of fluorescent particles was done using an Andor iXon Ultra EMCCD camera and 60x magnification objective. All images and movies were assembled for publication using NIS-Elements software (Nikon), Fiji/ImageJ, and Adobe Photoshop. For comparative analysis, fluorescence excitation intensity, exposure time, and other imaging parameters were consistent for all experimental conditions.

### Western blot analysis

Dissociated neurons or cell bodies from the S compartment were lysed in radioimmunoprecipitation assay (RIPA) buffer supplemented with 1 mM dithiothreitol (DTT) and protease inhibitor cocktail (Sigma-Aldrich). Lysates were incubated on ice for 30 min, sonicated, and centrifuged at 11,000 rpm at 4°C. Supernatants were transferred into new tubes and mixed with 5x Laemmli buffer. Samples were heated at 90°C for 5 min before resolved by 4–12% NuPAGE BisTris gels (Invitrogen). Proteins were transferred to nitrocellulose membranes (Whatman) using semidry transfer (Biorad). For blocking, membranes were incubated in 5% non-fat dry milk in phosphate-buffered saline (PBS) solution for 1 h at room temperature. Immunoblots were performed using primary and secondary antibodies diluted in 1% milk PBS solution. Membranes were incubated with chemiluminescent substrates (Supersignal West Pico or Dura, Thermo scientific). Protein bands were visualized by exposure on HyBlot CL (Denville scientific) blue X-ray films. Primary antibodies used in this study: Mouse monoclonal antibody (mAb) anti-β-actin (Sigma), anti-Us9 mouse mAb IA8 clone [51], anti-VP5 mouse mAb (gift of H. J. Rziha, Federal Research Center for Viruses Diseases for Animals, Tubingen, Germany), anti-UL47, anti-UL35 and anti-UL36 polyclonal rabbit sera [gifts of T. Mettenleiter, Friedrich-Loeffler Institut [52–54]], anti-Us3 mouse mAb [21], rabbit polyclonal anti-EP0 (gift of H. Kida, Hokkaido University) [55], anti-gD polyclonal rabbit sera (gift of K. Bienkowska-Szewczyk University of Gdansk), anti-cJun (Cell Signaling), anti-phospho-cJun (Ser63; Cell Signaling) anti-AKT (Cell Signaling), anti-phospho-AKT (Ser473; Cell Signaling), anti-PKA substrates (P-S/T Kinase substrate Ab Sampler, Cell Signaling). Horseradish peroxidase-conjugated secondary mouse or rabbit antibodies (KPL) were used at 1:10000 dilution.

### Quantitative RT-PCR

Q-RT-PCR was performed with Eppendorf Realplex Mastercycler. Reaction mixture was prepared using Kapa Syber Fast qPCR kit and samples were prepared as triplicates. Each experiment was done in duplicates. To determine genomic DNA amounts in neuronal soma, each S compartment was lysed in 10 μl of RIPA. 5 μl of this lysate was treated with proteinase K (New England Biolabs) for 50 min. at 55°C followed by 5 min at 95°C. To titer entry deficient PRV mutants (gBnull and gDnull), 90 μl of virus stock was first digested with 100 units of DNase I (Invitrogen) to remove contaminating DNA before proteinase K treatment. Viral genomic DNA was quantified by using UL54 specific primers as published [56]. PRV Becker nucleocapsid DNA and PRV Becker virus stock (1x10^8^pfu/ml) were used as standards to determine the amount of viral DNA corresponding to one plaque forming unit. To determine transcript amounts, total RNA was extracted from cell bodies in the S compartment using RNeasy Plus Mini kit (Qiagen). cDNA synthesis was performed using SuperScript III first-strand synthesis kit (Life Technologies) with Oligo-dT primers. LAT specific primers (fw: 5`-GATGCAGTCCAGACAG-3`, rev: 5`-GTAGTGGTCCCGAGTTGC-3`) amplifying a 141 bp fragment were used. EP0 transcript was quantitated using primers (fw: 5`-GGGTGTGAACTATATCGACACGTC-3`, rev: 5`-TCAGAGTCAGAGTGTGCCTCG-3`) to amplify a 150 bp region. Plotted values were calculated using the –ΔΔCt method and normalization to 28S rRNA (fw: 5`-GGGCCGAAACGATCTCAACC-3`, rev: 5`-GCCGGGCTTCTTACCCATT-3`). Fold changes were calculated using the comparative CT method (2^**(C**^_**TX**_^**-C**^_**TR**_^**)**^_**control-**_^**(C**^_**TX**_^**-C**^_**TR**_^**)**^_**test**_) where C_TX_ is the threshold cycle of the gene of interest and C_TR_ is the threshold cycle of the reference gene (28S rRNA) [57].

### Statistical analysis

One-way analysis of variance (ANOVA) with Tukey’s posttest or Mann-Whitney test was performed using GraphPad Prism 5.0. Values in the text, graphs, and figure legends throughout the manuscript are means ± standard errors of the means (SEM).

### Ethics statement

All animal work was performed in accordance with the Princeton Institutional Animal Care and Use Committee (protocols 1947–16). Princeton personnel are required to adhere to applicable federal, state, local and institutional laws and policies governing animal research, including the Animal Welfare Act and Regulations (AWA); the Public Health Service Policy on Humane Care and Use of Laboratory Animals; the Principles for the Utilization and Care of Vertebrate Animals Used in Testing, Research and Training; and the Health Research Extension Act of 1985.
